# A controlled trial of a co-designed virtual reality experience to support wellbeing in students

**DOI:** 10.1038/s41598-026-61827-6

**Published:** 2026-08-07

**Authors:** Aileen O’Brien, Baba Sheba, Rachel Simnett, Jared G. Smith, Nadia Mantovani, Sarah White, Simon Riches, Darren Bell, Michael Abbott, Alexandra Collisson, Trudi Edginton, Marco Gillies, Xueni Pan

**Affiliations:** 1 Population Health Research Institute, City St George’s University of London, London, SW17 ORE UK; 2 Simnett Consulting, Sudbury, UK; 3 Southwest London and St George’s Mental Health NHS Trust, London, UK; 4 Department of Psychology and Human Development, University of East London, London, UK; 5 Kings College London, London, UK; 6 South London and Maudsley NHS Foundation Trust, London, UK; 7 Queen Mary University of London, London, UK; 8 Goldsmiths University of London, London, UK

**Keywords:** Health care, Psychology, Psychology

## Abstract

Increasing rates of mental health problems among university students require innovative interventions. This study evaluated a multi-sensory, virtual reality (VR) intervention aimed at reducing stress and exam anxiety among students at a healthcare university in the UK. Participants (*n* = 131) were randomised to either the VR condition or the same experience on their smartphones as Video, with both groups engaging over five consecutive days. Pre- and post-session measures were collected for perceived stress, well-being, and depressive symptoms, alongside qualitative assessments of the experiences. Both VR and Video participants reported statistically significant reductions in perceived stress levels at follow-up. Immediate positive affective changes were greater in the VR than Video condition, with high levels of immersion, which correlated with improved emotional outcomes. Themes within the qualitative data highlighted the therapeutic impact of VR, with participants expressing increased calmness and concentration. These findings suggest that this intervention may serve as an effective and engaging tool to reduce student stress and exam anxiety. This study contributes to the growing literature on digital mental health and emphasises the need for institutions to explore immersive technologies as part of mental health support services. Further research is warranted to determine long-term efficacy and optimal usage strategies for VR in academic environments.

**Supplementary Information:**

The online version contains supplementary material available at https://doi.org/10.1038/s41598-026-61827-6.

## Introduction

There is a well-documented crisis in young people’s mental health. Between 2016/17 and 2022/23, the proportion of students across UK universities experiencing mental health difficulties increased from 6 to 16%^1^, becoming an urgent concern for public health and policy^2,3^. Depression and anxiety are the most common mental health problems among students^4^. Associated factors include financial stress, social isolation and loneliness, identity and self-esteem issues, lack of access to support, transition challenges, and academic pressure^5^.

In specialist fields, such as medicine, a higher prevalence of mental health difficulties has been documented in students^6^, who are more likely to experience a range of problems, including depression, anxiety and burnout that can contribute to higher levels of exam stress^7,8^. There has been a growing demand for support services^9,10^. However, only a third of students with mental health problems seek support from counselling services^11^ with many medical students not seeking any help^8^, actively avoiding treatment^12,13^ or being unwilling to disclose mental health concerns^13,14^. Internationally it is accepted that young people’s mental health has been in decline since the pandemic and it has been postulated that geopolitical anxiety and uncertainty coupled with heavy smartphone and social media use may be contributory factors^15^.

Virtual Reality (VR) has been shown to be a promising tool to promote relaxation and reduce stress in both clinical and non-clinical populations^16,17^ with increased interest in its use for support in the general population, patients and students^16–21^. Recent systematic reviews highlight that VR is an engaging and accessible intervention for students and young adults with demonstrated efficacy for stress reduction. These systematic reviews recommend inviting students to contribute to further refinement of immersive environments that align with established wellbeing approaches^19,20,22^.

Virtual reality (VR) hypnotherapy is an emerging intervention that integrates immersive digital environments with established hypnotic techniques. By enhancing presence and multisensory engagement, VR is felt to facilitate deeper absorption^23^.

Early evidence suggests that VR-assisted hypnosis may be particularly beneficial in pain management, anxiety disorders, and procedural distress. The mechanism is thought to involve attentional modulation and distraction, combined with hypnotic suggestion, which together alter pain perception and emotional processing with few adverse effects^24^. The main reported side effect of VR interventions is ‘cybersickness’, similar to motion sickness^25^. Thus, it is important to differentiate between the potential overlap of the somatic symptoms of anxiety and the cybersickness symptoms associated with VR.

Our research team evaluated a novel multi-sensory hypnotherapy-based VR intervention developed by the company Phase Space, in a feasibility study with 15 healthcare students, reporting significant improvements in wellbeing^26^. This informed the further development of this immersive multi-sensory VR intervention co-designed with 36 university students, with student wellbeing and exam stress in mind^27^. These were one-on-one interview sessions with students, with twelve students in three rounds, giving feedback about both the design and content. There was a mix of exploratory questions around context and appetite with focussed usability questions to refine the experience.

The mixed-methods Randomised Controlled Trial (RCT) was designed to establish whether the VR intervention delivered over 5 consecutive days could reduce subjective levels of stress, anxiety and depression and improve wellbeing in students compared to delivery of the same content on mobile phones. It took place with students in a university setting with baseline, post intervention and 2-week follow up measures. We hypothesised that enhanced immersion in VR compared to mobile phone delivery will mediate superior psychological outcomes through increased sensory engagement and presence.

## Materials and methods

### Design

The study employed a partially blinded, parallel randomised controlled trial (RCT) design. A clinically informed multi-sensory hypnotherapy-based experience using an immersive VR app with headphones was compared with the same experience watched on a smartphone and listened to through headphones (Video condition). This was delivered over five days with a two-week follow-up.

### Participants

The study took place at St George’s, University of London (SGUL), the only specialist healthcare university in the UK, with 5500 full-time students on a range of healthcare courses: medicine, physiotherapy, diagnostic and therapeutic radiography, physician associate and paramedic science. All enrolled students were offered the opportunity to take part in the experimental study exploring the effects of the intervention designed to reduce stress and exam anxiety and were recruited over a 1-month period (Oct–Nov 2023). They were informed via the Student Union (SU) social media and invited to register on the study website. Students with a history of seizures or a pacemaker were excluded due to possible risks of immersive VR. There were no other exclusion criteria; a deliberate decision was made not to exclude students with mental health concerns.

**Fig. 1:**
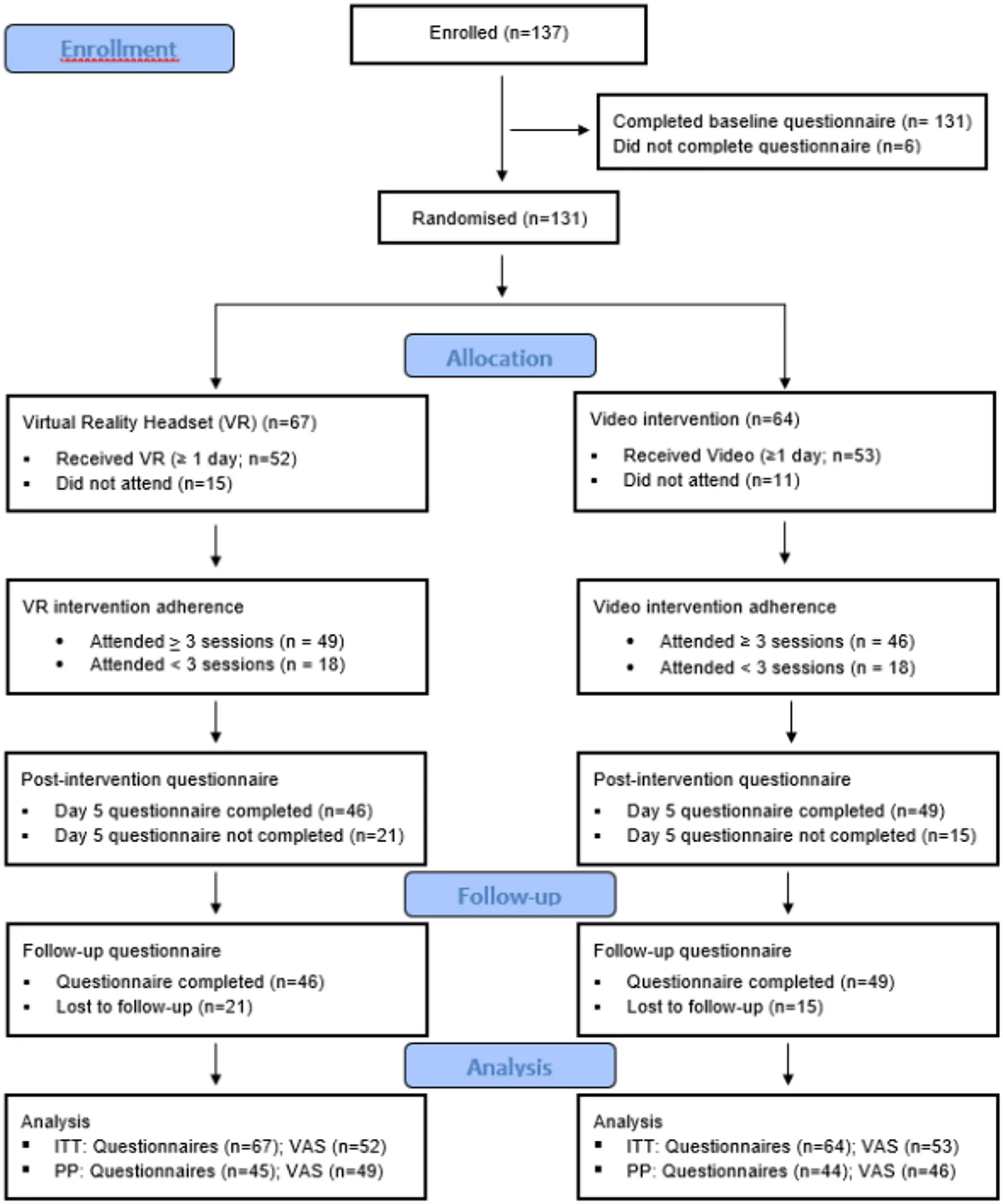
Information sheet given to participants to explain the stages of the Virtual Reality Headset experience. Consolidated Standards of Reporting Trials (CONSORT) flow chart for trial recruitment. Note: only 42 VR and 41 Video participants completed the Simulation Sickness Questionnaire. ITT, intention to treat; PP, per protocol.

131 participants were recruited and randomly allocated to VR (*n* = 67) or Video (*n* = 64) with CONSORT trial recruitment data reported in Fig. 1. The randomisation schedule was generated using freely available software (Sealed Envelope) by SW, using randomly permuted blocks sizes of 4, 6 or 8 without stratification. Participants could not be blind to the intervention but the researchers were blind to the data at the stage of analysis. After randomisation students were emailed with an ID number and asked to come to the Student Union where they were led to one of two rooms. All interventions took part in the Student Union and the headsets were kept there for the duration of the study. Participants received £60 vouchers as remuneration.

A facilitator demonstrated the use of the Meta Quest 2 VR headset, then the students took part in the VR experience. QR codes were available for students to access the Video experience and to complete numerical analogue scales and standardised questionnaires pre and post intervention for the 5-day trial.

### Intervention and control conditions

The intervention arm consisted of a daily VR hypnotherapy-based experience lasting approximately 7 min combining soothing visuals, sounds and music with pre-recorded expert guidance curated by an experienced therapist. It combines multisensory stimuli and positive suggestions aiming to reduce exam anxiety, create a sense of calm and provide synchronicity between the mind and the body^28,29^. The audio is augmented with generative VR graphics beginning in a domed hall. The user is gently guided through a transition into darkness before rings of brightly shining particles form and travel towards the viewer, passing around them on all sides before fading. The synchronicity and timing of these particle rings combined with gentle pulses aid the therapist’s suggestions to slow the breath, reduce tension in the body and calm the mind. The voiceover incorporates positive suggestions of coping mechanisms for dealing with work stress and exams. These varied slightly each day. The user is gradually brought back to the domed hall, grounded in the present and asked to remove their headset. The active control arm consisted of the same daily experiences that were delivered by smartphone and listened to with headphones without an immersive component.

### Ethics

All participants provided written informed consent. Anonymity and confidentiality were guaranteed. The study received ethical review and approval from the St George’s University of London Research Ethics Committee Ethics reference: 2023.0136.

### Measures

#### Demographic information

At baseline, participant information was collected relating to age, sex, gender and mental health service use over the last month.

### Momentary negative and positive affective states

A 10-point numerical analogue scale (VAS), with anchors on extreme values, was selected to assess the immediate effect on level of negative and positive affective states (“stress”, “calm”, “happy”, “sad”, and “anxiety”) before and after each intervention accessed via the QR codes. These momentary assessment items have been widely used in previous studies^29–31^ including VR interventions^31,32^. Participants’ level of immersion, operationalised as the subjective sense of presence within the virtual environment, was also evaluated on a 10-point VAS scale after each session.

### Cybersickness

The Simulator Sickness Questionnaire (SSQ) was administered before and after the intervention on the final session. It is intended to evaluate cybersickness symptoms and is regarded as the “gold standard”^33^. It consists of three subscales (nausea, oculomotor, and orientation), with higher scores indicating more severe symptoms. The Cronbach α for the scale was 0.834 at pre-session and 0.707 at post-session.

### Short-term outcomes in perceived stress, wellbeing, and depressive symptoms

To assess changes in perceived stress, wellbeing, and depressive symptoms, participants completed standardised questionnaires at baseline and at 2 weeks post-trial with investigators blinded to experimental condition. Global perceived level of stress was measured with the 10-item version of the Perceived Stress Scale (PSS-10)^34–36^ whereby participants rate how unpredictable, uncontrollable and overloaded they have found their life over the past month on a 5-point VA scale (from 0= ‘never’ to 4= ‘very often’). Higher scores indicate greater stress levels, with scores ≥ 27 considered high perceived stress. Internal consistency was high in our sample (baseline administration; Cronbach α = 0.882).

Positive states of being, thinking, behaving and feeling in the last 2 weeks were assessed via the Warwick-Edinburgh Mental Wellbeing Scale (WEMWBS)^37^. This comprises 14 statements with five response categories (from 1 = ‘none of the time’ to 5 = ‘all of the time’). Total scores range from 14 to 70 with scores ≤ 42 indicative of low wellbeing. Internal consistency was high in the current sample (baseline administration; Cronbach α = 0.914).

Depression symptoms were measured using the Patient Health Questionnaire-2 (PHQ-2)^38^ a validated screening measure for a depressive disorder that yields total scores from 0 to 6 with a score ≥ 3 being a positive screen for a depressive disorder.

### Intervention satisfaction

At the end of the trial, participants were asked which group they would have preferred to be in, having been aware at the consent stage that there were two arms. Satisfaction with the intervention for participants completing VR or Video interventions was assessed via VAS, measuring levels of agreement (with anchors 1= ‘do not agree’, 10= ‘totally agree’) that the experience made them feel calmer, had a positive effect and is one they would recommend to other students. Participants were also asked to describe the impact the experience had on them personally, if it is something they could see themselves using, and to provide any other comments/feedback in an open-ended manner. At 2-week follow-up, participants rated levels of agreement (on VAS scales) on whether the intervention helped to manage stressful situations and could be a resource that they could see themselves using in the future.

### Sample size justification

The feasibility trial^26^ found within-group (Hedge’s g) effect sizes of 0.8 for change in wellbeing and depression. As the control condition is active, we would expect a lower between group effect size. Eighty-eight participants were required to detect a between group effect size (Cohen’s d) of 0.6 or greater, with 80% power at 5% significance level. To account for a 25% dropout rate, the target sample size was 120.

### Data analysis

Summary statistics were used to describe the sample with respect to demographics and baseline measures. Changes in perceived stress (PSS-10), wellbeing (WEMWBS) and depression symptoms (PHQ-2) over the 2-week study period were assessed with multilevel mixed-effects linear regression models (LMMs) using restricted maximum likelihood estimations. The LMMs specified fixed Condition (VR/Video) and Time (Baseline/Follow-up) main effects and a Condition × Time interaction (with Participant as a random effect) and yielded within- and between-group estimates of change. Immediate effects of the intervention for momentary affective states were analysed using a similar mixed model design. These LMMs included three fixed main effects, Condition, Pre/Post-session and Day^1–5^, and Condition*Pre-/Post-session, Condition*Day and Condition*Pre-/Post-session*Day interactions. This allowed examination of change within and across sessions according to Condition. Participant and Day were added as random effects. For Immersion, a mixed model design with fixed Condition and Day effects and a Condition*Day interaction was employed. In all LMMs, the most appropriate covariance structure for each model was determined by likelihood ratio tests and minimum values for the Akaike information criterion, and all participants with at least one data point were included. The LMM method yields unbiased estimates in the case of modest-sized samples with missing data, by assuming data are missing at random and is consistent with an ‘intention-to-treat’ (ITT) approach. Little’s Test indicated that missing short-term outcome (perceived stress, wellbeing and depressive symptoms) and momentary affective state data were missing completely at random; for all tests, *p* > 0.256.

Secondarily, per-protocol analyses were conducted, including only participants attending at least 3 of the sessions, and who provided follow-up data. Efficacy for treatment completers on VAS measures was assessed by calculating between-group differences for each domain using analysis of covariance (ANCOVA). Changes in perceived stress, wellbeing and depression symptoms over the study period were assessed using a 2 (Baseline, Follow-up) by 2 (VR, Video) mixed-measures analysis of variance (ANOVA). These approaches require a complete dataset, and as such, were appropriate for per-protocol analyses where measure completion is a necessary condition. Associations between within-session changes on VAS with levels of intervention immersion and trial condition and cybersickness (SSQ scores) were tested using t-tests, ANCOVA and partial correlations.

Where distribution of model residuals failed to approximate a Gaussian distribution (identified via examination of histograms, Q-Q plots and levels of skew/kurtosis), LMM and mixed-measures ANOVA were administered using Box–Cox transformed data while ANCOVA and correlational analyses employed bootstrapping. LMMs were implemented with heterogenous covariance structures and ANOVA/ANCOVA employed robust standard errors where heteroscedasticity was indicated (from plots of residuals by fitted values and Breusch–Pagan tests, respectively). Effect sizes (Hedge’s g) for adjusted pre-to-post session differences were calculated for participants within each condition by dividing the difference between (adjusted) mean scores at the time points by the pre-session SD. Between-group effect sizes were calculated by dividing the difference between VR and Video intervention participants’ (adjusted) pre-to-post-treatment mean change by the pooled pre-session SD. The False Discovery Rate^39^ with control set at 5%, was applied to manage the increased risk of false-positive results when (multiple) tests were performed across VAS, SSQ and satisfaction measures (for each set of analyses); otherwise, the criterion for statistical significance set at *p* < 0.05. Statistical analyses were undertaken using SPSS Version 29, supplemented where required by Stata SE Version 16.0.

### Qualitative methods and analysis

The open text data was exported from Excel files into NVivo qualitative data analysis software version10 (QSR International Pty Ltd) and all names were anonymised. Rapid qualitative analysis was selected^40^, as a rigorous, pragmatic and applied methodology to analyse brief, structured qualitative interview data and capture the experiential and contextual aspects of the intervention within and across the identified themes.

The following steps were taken (i) reading the collected data multiple times to compile and make sense of the categories within it, (ii) identifying codes that captured key words in the data, (iii) coding the remaining data by assigning codes to the new words or content identified that fit into existing codes, (iv) grouping the codes and organising them into categories and subcategories based on how they related to one another, (v) reviewing individual themes by reading all the collated extracts of each theme to develop a coherent and meaningful alignment across the themes. The qualitative research team comprised researchers with backgrounds in psychiatry, psychology, sociology of health, and mental health. The authors acknowledge that their prior experience and knowledge of the study topic could influence data interpretation. To enhance reflexivity, coding decisions and theme development were discussed among the research team, and interpretations were reviewed and refined through analytical discussions to ensure that findings remained grounded in participants’ accounts.

## Results

### Trial recruitment, intervention adherence and measure completion

**Fig. 2:**
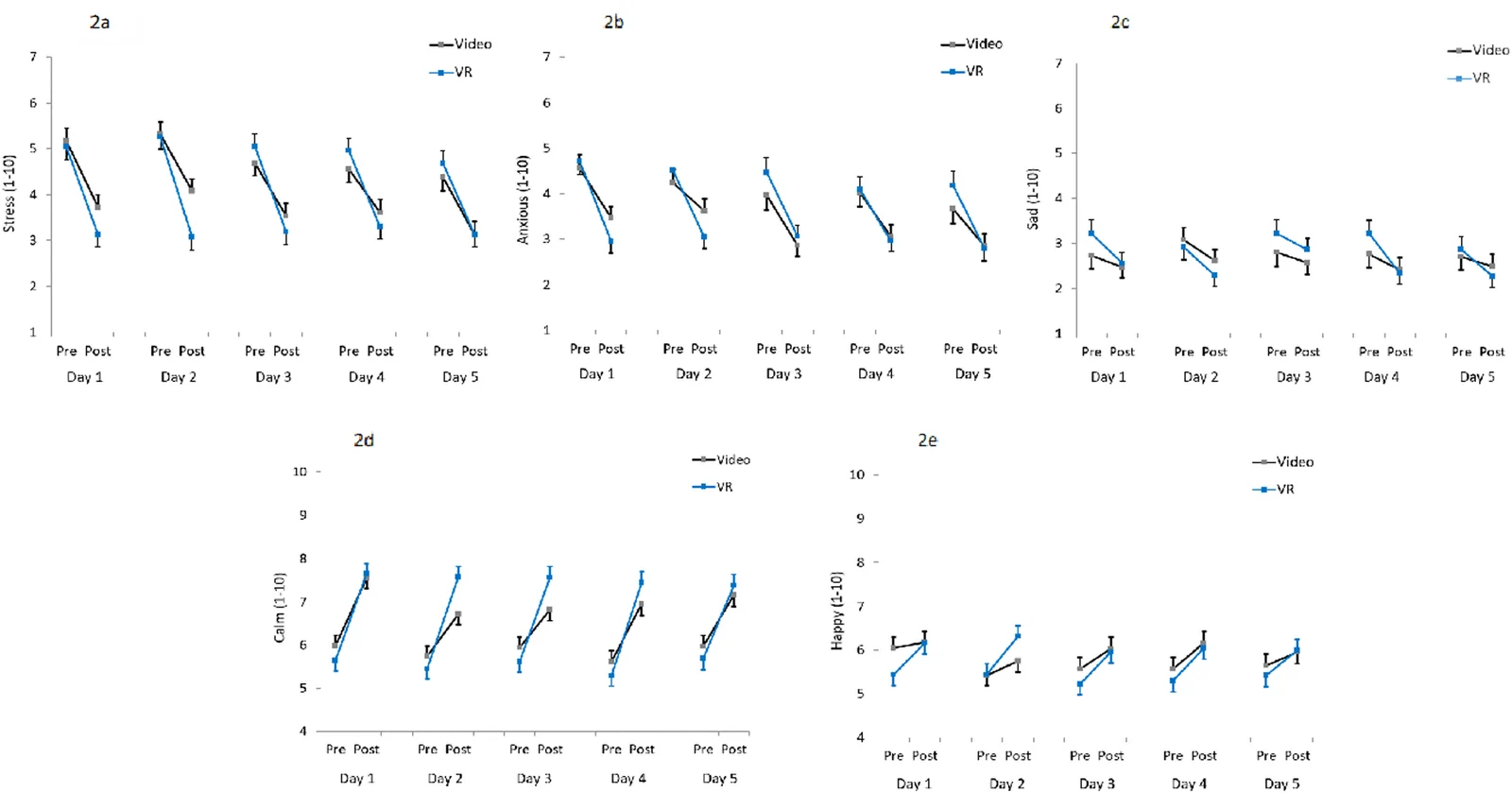
Consolidated Standards of Reporting Trials (CONSORT) flow chart for trial recruitment and intervention adherence. Note: only 42 VR and 41 Video participants completed the Simulation Sickness Questionnaire. ITT, intention-to-treat; PP, perprotocol. (**a**–**e**) Adjusted pre- and post-session mean (SE) VAS values in each study session (Day) for VR Headset (VR) and Video intervention study participants (ITT approach; 2a = Stress; 2b = Anxious; 2c = Sad; 2d = Calm; 2e = Happy).

137 student participants were enrolled of whom 131 consented to participate, completed baseline questionnaires, and were allocated to a trial arm, VR = 67 and Video = 64 (Fig. 2). Twenty-six participants (19.8%) did not attend any intervention session (VR *n* = 18, 26.9%; Video *n* = 11, 17.2%) and were not included in short-term outcome analyses. Of the 67 participants randomised to VR, 49 (73.1%) attended at least 3 (of 5) sessions as opposed to 46 (71.9%) participants randomised to the Video intervention. (Fig. 2). Post-intervention and follow-up questionnaire data were available for 95 participants (72.5%) (VR *n* = 46, 68.7%; Video *n* = 49, 76.6%), although these were not the same participants at each assessment. Non-attendance of (any) sessions, intervention adherence and post-intervention and follow-up questionnaire completion were comparable between conditions (for all Chi-square comparisons, *p* > 0.310).

### Participant demographic and baseline characteristics

Demographic data and pre-intervention levels of perceived stress, wellbeing and mood of participants are shown in Table 1. Two-thirds of participants were female, and the mean age was almost 22 years (median = 20.0, IQR = 19.0, 23.0). On average, participants reported moderate perceived stress. A little more than a quarter of participants screened positive for depression and almost 40% indicated low wellbeing. A quarter of participants had used a mental health service in the last month; most of whom reported using a self-help app. There was little indication participants’ age, sex or use of mental health services over the last month were linked to non-attendance of sessions, intervention adherence, post-intervention questionnaire completion or follow-up questionnaire completion (for all Chi-square/t-test comparisons, *p* > 0.154).

**Table 1:** Demographics and pre-intervention scores for perceived stress, wellbeing and mood measures in Virtual Reality Headset (VR; *n* = 67) and Video (*n* = 64) intervention groups.

	Total	VR	Video
	*n* (%)	*n* (%)	*n* (%)
Gender			
Female	86 (65.6)	47 (70.1)	39 (60.9)
Male	44 (33.6)	19 (28.4)	25 (39.1)
Non-binary	1 (0.8)	1 (1.5)	0 (0.0)
	Mean (SD)	Mean (SD)	Mean (SD)
Age (years)	21.84 (5.31)	22.36 (6.64)	21.30 (3.38)
PSS (0-40)	20.92 (6.96)	22.18 (6.29)	19.59 (7.42)
WEMWBS (14-70)	45.25 (9.22)	44.40 (8.30)	46.14 (10.77)
PHQ-2 (0-6)	1.87 (1.64)	1.96 (1.51)	1.78 (1.77)
	n (%)	n (%)	n (%)
High stress (PSS≥27)	30 (22.9)	15 (22.4)	15 (23.4)
Low wellbeing (WEMWBS≤42)	51 (38.9)	29 (43.3)	22 (34.4)
Depression (screen; PHQ-2≥3)	34 (26.0)	18 (26.9)	16 (25.0)
	n (%)	n (%)	n (%)
Mental health service use (over last month)	33 (25.2)	20 (29.9)	13 (20.3)
University counselling	9 (6.9)	4 (6.0)	5 (7.8)
Primary care (GP)	7 (5.3)	5 (7.5)	2 (3.1)
Secondary care	6 (4.6)	5 (7.5)	1 (1.6)
IAPT	3 (2.3)	2 (3.0)	1 (1.6)
Private counselling	3 (2.3)	2 (3.0)	1 (1.6)
Self-help app	19 (14.5)	10 (14.9)	9 (14.1)

PSS, Perceived Stress Scale; WEMWBS, Warwick-Edinburgh Mental Wellbeing Scale; PHQ-2, Patient Health Questionnaire-2; GP, general practitioner; IAPT, Improving Access to Psychological Therapies.

### Efficacy of VR: intention to treat analyses

Following baseline assessment and randomisation, we analysed PSS-10 using linear mixed models incorporating both intent-to-treat and per-protocol approaches. The estimated marginal means (and 95% confidence intervals CI)) obtained from LMMs (Table 2) reflected a highly significant decrease in participants’ perceived stress over the course of the study (main effect of Time: beta = 3.11, CI = 1.63,4.59; *p* = 0.001), with within-group decreases significant for participants in each condition (VR g = 0.48, CI = 0.26,0.72; Video g = 0.20, CI = 0.01,0.39). There was a trend for (overall) wellbeing scores to increase from pre-intervention to follow-up (*p* = 0.092), but no indication of an improvement in depression symptoms (*p* = 0.924). There was no differential treatment benefit of the VR application over the Video intervention for perceived stress, wellbeing and mood (Table 2 VR vs. Video participants Pre-to-Post change; for all interactions between Condition and Time *p* > 0.123). There was no main effect of Condition for PSS-10 (*p* = 0.456) or WEMWBS (*p* = 0.635). While there was a significant effect of Condition on PHQ-2 scores (beta = − 0.59, CI = − 1.20, − 0.02; *p* = 0.037), when the model was readministered, controlling for baseline PSS-10 scores (which were higher in VR participants at baseline), this was no longer significant (*p* = 0.367). Including baseline PSS-10 score as a covariate in the LMM concerning WEMWBS scores did not change the (null) findings.

### Immediate effect of the VR and video interventions

**Fig. 3:**
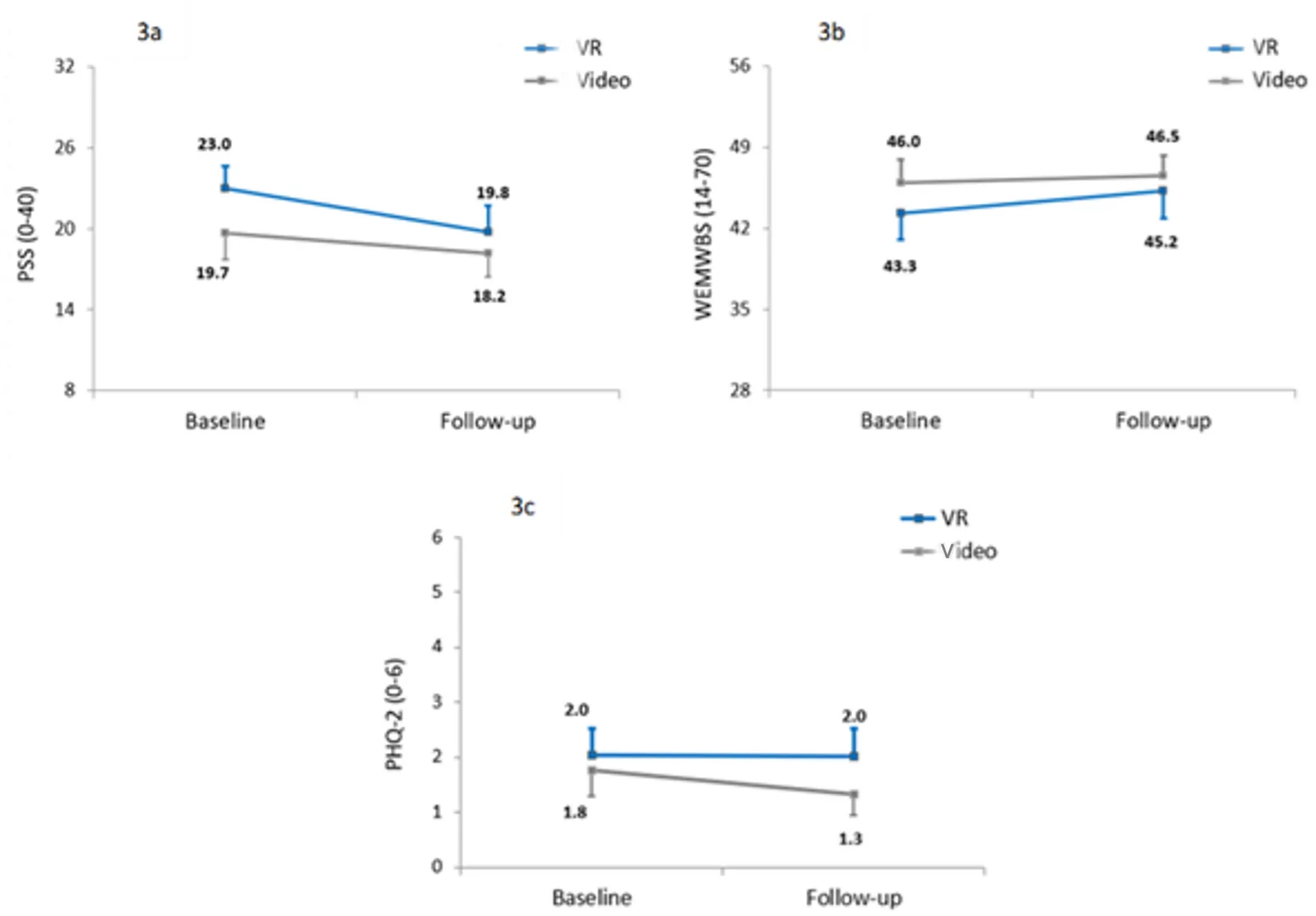
Adjusted pre- and post-session mean (SE) momentary affective visual analogue scale (VAS: 1–10) and Immersion (1-10) values in each study session (Day) for Virtual Reality Headset (VR) and Video intervention study participants. Intention-to-treat (ITT) approach; 3a = Stress; 3b = Anxious; 3c = Sad; 3d = Calm; 3e = Happy.

Session scores (estimated marginal means from LMMs) on the emotion VAS are shown in Fig. 3a–e with overall (pre- and post-session) scores shown in Table 3. There were significant interactions between Condition and Pre-/Post-session in Stress, Anxious, Calm and Happy domains (for all, *p* < 0.031), signifying greater immediate benefits in the VR condition compared to the Video intervention (g ranged from 0.24 to 0.46; see Table 3). While there was a trend for sad to decrease more for participants receiving VR than the Video intervention, the difference was not significant (*p* = 0.061). For each VAS, analysis revealed a highly significant main effect of Pre-/Post-session (for all, *p* < 0.001; pre-to-post session changes within both VR and Video groups were significant for all measures; see Table 3) across the overall study sample, but no main effect of Condition (for all, *p* > 0.158). There were significant main effects of Day for Stress and Anxious domains (for both, *p* < 0.027), reflecting decreases in each over the course of the 5 sessions. No main effect of Day was observed in Calm, Happy or Sad domains (for all, *p* > 0.075), and there were no significant interactions between Condition and Day or between Condition, Day and Pre-/Post-session on any of the VAS measures. Significant findings remained unchanged when baseline PSS-10 scores (which differed between conditions) were controlled for in LMMs. Exploratory (descriptive) analyses that (separately) stratified groups according to the presence of high perceived stress (PSS-10), low well-being (WEMWBS) and positive screen for depression (PHQ-2) at baseline (by adding a main effect and interaction term (with Condition*Pre-/Post-session) in the LMMs) indicated improved (within-group) momentary affective states were, in general, of comparable magnitude in VR participants irrespective of baseline status but often higher in Video participants with high stress, low wellbeing and positive depression screen relative to those with low/moderate stress, medium/high wellbeing and negative depression screen (Tables S1–S3).

**Table 2:** Adjusted mean values for perceived stress, wellbeing and mood at pre-intervention and follow-up with within- and between-group change for Virtual Reality Headset (VR) and Video intervention participants with estimates of within-group and between-group changes (ITT approach).

	PSS-10 (0-40)		WEMWBS (14-70)		PHQ-2 (0-6)	
	Mean (CI)	Hedge’s g (CI)	Mean (CI)	Hedge’s g (CI)	Mean (CI)	Hedge’s g (CI)
VR						
Pre-intervention	22.18 (20.54,23.82)		44.40 (42.25,46.55)		1.96 (1.58,2.34)	
Follow-up	19.07 (17.26,20.89)		46.10 (43.71,48.48)		1.95 (1.52,2.38)	
Pre-to-follow-up change	3.11 *** (1.64,**4.57)**	**0.48 (0.26**,**0.72)**	1.70 (− 0.27,3.66)	0.20 (− 0.03,0.44)	0.004 (− 0.39,0.40)	0.01 (− 0.25,0.27)
Video						
Pre-intervention	19.59 (17.92,21.27)		46.14 (43.94,48.34)		1.78 (1.39,2.17	
Follow-up	18.11 (16.31,19.90)		46.91 (44.54,49.27)		1.36 (0.94,1.79)	
Pre-to-follow-up change	1.49 * (0.05,**2.92)**	**0.20 (0.01**,**0.39)**	0.77 (− 1.15,2.68)	0.08 (− 0.11,0.27)	0.42 * (0.04,**0.81)**	**0.23 (0.01**,**0.45)**
VR vs. video						
Pre-to-follow-up change	1.62 (− 0.45,3.68)	0.23 (− 0.11,0.58)	0.93 (− 1.83,3.69)	0.10 (− 0.24,0.44)	− 0.42 (− 0.97,0.14)	− 0.24 (− 0.58,0.11)

ITT, intention to treat; PSS-10, Perceived Stress Scale-10; WEMWBS, Warwick-Edinburgh Mental Wellbeing Scale; PHQ-2, Patient Health Questionnaire-2; Mean values are estimated marginal means from Linear Mixed Model (LMM) analyses. CI = 95% confidence intervals. Positive change scores and effect sizes represent an improvement for within-group values (VR participants, Video participants) and greater improvement for VR than Video participants for between-group values (VR vs. Video participants). Asterisks indicate significant differences between study conditions (within group Pre-intervention to Follow-up comparisons were corrected for multiple testing): *p < 0.05, **p < 0.01, ***p < 0.001. LMM analyses for PHQ-2 scores were calculated using transformed data; raw (adjusted) mean (change) values and effect sizes are shown.

### Immersion

Participants in the VR condition showed high levels of immersion. LMM analysis indicated VR participants experienced significantly greater immersion than participants receiving the Video intervention (main effect of Condition; g = 1.44 (CI 1.24,1.65; *p* = 0.001). But there was no main effect of Day or Condition*Day interaction, suggesting the advantage for VR participants remained largely the same across the trial sessions. Mean (post-session) immersion scores were more closely linked to mean change on emotional VAS measures (adjusted for pre-session scores) for participants in the VR condition (range *r* = 0.29 to 0.64) than those receiving the Video intervention (range *r* = 0.09 to 0.48; Table S4).

### Efficacy of VR: per-protocol analysis

Per-protocol (mixed model ANOVA) analyses yielded no significant interaction between Time and Condition for perceived stress, wellbeing or depression symptom measures (*p* > 0.111; for descriptive data, see Fig. S1a–c).There were significant effects of Time (F(1,87) = 17.80; *p* < 0.001) and Condition (F(1,87) = 4.23, *p* = 0.043) on perceived stress, suggesting participants who completed the intervention decreased perceived stress over the study period and that levels were generally higher for participants in the VR condition (largely due to differences at baseline); post-hoc analyses indicated VR participants significantly decreased PSS-10 scores at follow-up (mean difference (CI) = 3.24 (1.65,4.84); *p* < 0.001, g = 0.57, CI = 0.26,0.88) whereas participants completing the Video intervention did not (mean difference (CI) = 1.46 (− 0.12,3.03); *p* = 0.069, g = 0.21, CI = − 0.08,0.51). The estimated minimal clinically important change for the PSS-10 is 11 points for absolute change and 28% for relative change^36^. Seven participants at follow-up improved according to the former criterion, distributed equally across conditions (VR = 4 (8.9%), Video = 3 (6.8%); Chi-square *p* = 0.717) while more VR participants demonstrated relative MCIC (VR = 13 (28.9%), Video = 7 (15.9%); Chi-square *p* = 0.142), although differences were not significant. ANCOVA analyses indicated that overall (adjusted) VAS change scores were significantly greater for participants experiencing VR than those receiving the Video intervention in the domains of Stress (g = 0.35, CI = 0.09,0.62), Anxious (g = 0.26, CI = 0.07,0.46), Calm (g = 0.52, CI = 0.15,0.88) and Happy (g = 0.28, CI = 0.04,0.53) (for descriptive data, see Fig. S2). Post-hoc analyses considering each session separately indicated that differences were more marked in early sessions, most obviously Day 1 (Anxious) and Day 2 (Stress, Anxious, Calm; see Fig. S3a–d).

**Table 3:** Adjusted mean values for pre- and post-session momentary affective visual analogue scale measures (VAS: 1–10) with estimates of within- and between-group change for Virtual Reality Headset (VR) and Video intervention participants (ITT approach).

	Stress		Anxious		Sad		Calm		Happy		
	Mean (CI)	Hedge’s g (CI)	Mean (CI)	Hedge’s g (CI)	Mean (CI)	Hedge’s g (CI)	Mean (CI)	Hedge’s g (CI)	Mean (CI)		Hedge’s g (CI)
VR											
Pre-intervention	5.00 (4.59,5.41)		4.40 (3.93,4.86)		3.09 (2.62,3.56)		5.53 (5.20,5.87)		5.37 (4.98,5.75)		
Post-intervention	3.16 (2.75,3.58)		2.96 (2.58,3.35)		2.47 (2.11,2.83)		7.52 (7.19,7.86)		6.10 (5.72,6.48)		
Pre-to-post change	1.84*** (1.55,2.13)	0.95 (0.80,1.10)	1.44*** (1.16,1.71)	0.71 (0.57,0.84)	0.62*** (0.39,0.86)	0.27 (0.16,0.37)	1.99*** (1.65,2.34)	1.12 (0.93,1.31)	0.73*** (0.50,**0.96)**		**0.43 (0.29**,**0.56)**
Video											
Pre-intervention	4.83 (4.41,5.25)		4.09 (3.62,4.57)		2.82 (2.34,3.29)		5.85 (5.50,6.19)		5.65 (5.26,6.04)		
Post-intervention	3.61 (3.19,4.03)		3.17 (2.78,3.56)		2.51 (2.15,2.88)		7.04 (6.69,7.38)		6.02 (5.62,6.41)		
Pre-to-post change	1.22*** (0.92,1.51)	0.57 (0.43,0.70)	0.92*** (0.64,1.20)	0.42 (0.29,0.54)	0.30* (0.06,0.54)	0.14 (0.02,0.27)	1.19*** (0.84,1.54)	0.68 (0.48,0.88)	0.36** (0.13,**0.60)**		**0.21 (0.07**,**0.34)**
VR vs. video											
Pre-to-post change	0.62** (0.22,1.03)	0.31 (0.12,0.49)	0.52** (0.13,0.90)	0.24 (0.06,0.42)	0.32 (− 0.01,0.65)	0.15 (− 0.02,0.33)	0.80** (0.31,1.29)	0.46 (0.37,0.64)	0.37* (0.04,**0.69)**		**0.21 (0.03**,**0.39)**

ITT, intention to treat; Mean (change) values are estimated marginal means from Linear Mixed Model (LMM) analyses. CI = 95% confidence intervals. Positive change scores and effect sizes represent an improvement for within-group values (VR participants, Video participants) and greater improvement for VR than Video participants for between-group values (VR vs. Video participants). Asterisks indicate significant differences between study conditions (corrected for multiple testing across between-condition and within-condition comparisons): *p < 0.05, **p < 0.01, ***p < 0.001. LMM analyses for the Sad domain were calculated using transformed data; raw (adjusted) mean (change) values and effect sizes are shown.

### Changes of SSQ scores before and after VR and video interventions

Among all participants robust decreases in total SSQ score and each SSQ subscale were observed suggesting that adverse cybersickness effects of VR are not inevitable and that the intervention was effective at reducing somatic symptoms of cybersickness and anxiety within the seated controlled environment (Table 4). Within-group analyses (t-tests) revealed participants in both conditions evidenced reduced nausea, oculomotor disturbances and disorientation following the intervention (for all, *p* < 0.040); all between-condition (ANCOVA) comparisons of (adjusted) SSQ total/subscale change scores were non-significant (*p* > 0.216). Changes in SSQ domains were not significantly associated with (adjusted) mean pre-to-post session change on VAS measures in either group.

**Table 4:** SSQ before and after exposure to intervention in participants in Virtual Reality Headset (VR; *n* = 45) and Video (*n* = 41) conditions.

	Nausea	Oculomotor	Disorientation	Total
	Mean (CI)	Mean (CI)	Mean (CI)	Mean (CI)
VR participants				
Pre-session	20.78 (14.19,28.62)	30.49 (22.57,38.41)	26.60 (16.33,38.52)	30.34 (20.04,39.74)
Post-session	11.24 (7.37,15.50)	19.20 (14.28,24.12)	14.54 (9.69,19.83)	17.79 (12.89,22.69)
Pre-to-post change	9.54** (4.77,14.71)	11.29** (4.43,18.15)	12.06* (4.41,21.03)	12.55*** (5.24,19.86)
Video participants				
Pre-session	17.92 (13.74,22.62)	28.29 (22.04,34.53)	21.39 (14.95,28.26)	26.73 (20.81,32.79)
Post-session	10.94 (7.42,14.64)	21.08 (15.48,26.68)	16.30 (9.99,23.33)	19.06 (13.88,24.68)
Pre-to-post change	6.98** (3.82,10.35)	7.21** (3.10,11.32)	5.09* (1.08,9.39)	7.66** (4.11,11.38)

SSQ, Simulator sickness questionnaire. Within-group (pre-to-post-intervention) change; Asterisks indicate significant differences after correction for multiple testing: *p < 0.05, **p < 0.01, ***p < 0.001.

### Satisfaction with intervention

At post-intervention, all but 2 of 45 (4.4%) participants completing the Video intervention stated a preference for allocation to the VR application. All participants receiving the VR intervention indicated VR as their preference. Furthermore, compared with participants receiving the Video intervention (independent-group t-tests), those in the VR condition had significantly higher levels of agreement at post-intervention that the experience made them feel calmer (*g* = 0.81, CI = 0.38,1.23), had a positive effect on themselves (*g* = 0.69, CI = 0.27,1.10) and that they would recommend to other students (*g* = 0.84, CI = 0.27,1.41). At follow-up, those in the VR condition had higher levels of agreement that taking part in the trial had helped them to manage exam stress and stressful situations (*g* = 0.67, CI = 0.11,1.24) and is a resource they could see themselves using in the future (*g* = 0.79, CI = 0.22,1.36).

### Qualitative analysis

Participant responses within the open text data were anonymised, coded and analysed using NVivo software. The themes were reviewed and refined with a selection of excerpts to reflect individual experiences and alignment across the extracts. Five main themes emerged from the qualitative analyses reflecting a range of therapeutic effects of VR technology with 89% of VR group (42 out of 47) reporting experiencing a positive effect on their well-being as evidenced across the students’ qualitative responses that are briefly summarised below: (1) Immediate improvements in mental and physiological states (2), Enhanced sense of control, confidence, and agency (3), Intentional structured self-care practices (4), Broader and sustained impact on wellbeing (5), Limited impact of non-immersive video.

1. Immediate improvements in mental and physiological states.

This theme captures how the VR functioned as a powerful tool for immediate emotional regulation, enabling students to reduce perceived stress, achieve a state of calm, and regain psychological equilibrium during academically demanding periods. Beyond transient relaxation, the VR facilitated a broader reset of cognitive and emotional states, enhancing students’ capacity to re-engage with revision tasks.

Psychological reset: A prevailing subtheme across the dataset was the capacity of VR to facilitate changes in the cognitive appraisal of stress that reduced cognitive load and improved mental clarity and composure.

Feeling more grounded: This subtheme reflected the descriptions of a rapid transition from physiological states of stress, overwhelm and tension to interoceptive awareness, feelings of calmness and ability to self-soothe for the majority of participants following each VR session.

Student excerpts highlighted the additional benefits when feeling under pressure particularly during exam periods.

*“I think doing it so regularly grounded me during a particularly stressful week and made me feel as if my goals were more attainable.”* (VR group).

*“It has grounded me daily and helped me to escape the stress of revising but has still been productive as it helps to reset me.”* (Video group).

*“I felt lighter*,* calmer and had fewer negative thoughts running through my head.”* (Video group).

*“Whenever I had finished my session*,* I felt remarkably calmer and relaxed.”* (VR group).

2. Enhanced sense of control, confidence, and agency.

This theme captures how VR, and to a lesser extent Video, fostered a strengthened sense of personal control, self-efficacy, and confidence, enabling students to approach academic challenges with increased assurance.

Emotional stability: this subtheme reflected the reports from some students of a reduced tendency to fluctuate between extreme emotions associated with stress and/or disengagement.

“I am in control”: this subtheme captured the internalisation of the affirmations that fostered an increase in confidence in managing workload and academic demands.

Here, students emphasise how the intervention reinforced a sense of control and confidence, describing these effects as both reassuring and motivating.

*“I feel more in control. I’m really surprised*,* as I wasn’t expecting it to have such an effect.”* (VR group).

*“It boosted my self-confidence and made me believe in myself. It’s a voice telling me I can do it and that really helped.”* (VR group).

*“It is reassuring to hear external voices say the same things I try to tell myself.”* (VR group).

*“It regulated my emotions from being too high or too low.”* (VR group).

“*Although I am not prepared for my exams content wise*,* I am more willing and hopeful to attempt it.* (VR group)

Although less frequently reported, similar effects were also observed in the Video group, suggesting that even non-immersive interventions may contribute to perceptions of control, albeit to a lesser extent.

3. Intentional structured self-care practices.

This theme reflects how the interventions encouraged intentional self-care practices, grounding, and the use of structured pauses as a means of managing stress and maintaining psychological balance.

Dedicated space for self-care: this subtheme captured the students’ gratitude and acknowledgement of the intentional creation of time purely for themselves which was often lacking in their daily routines.

Pause, reflect, and reconnect: this subtheme highlighted the students’ experiences of enhanced self-awareness and their subsequent development of intentional mindful strategies to pause and connect with their thoughts, internal sensations and feelings.

Importantly, these pauses were not framed as avoidance but rather as a productive reset, enabling students to return to their work with improved focus and reduced stress. Self-care was conceptualised—not as indulgence, but as integral to academic performance. Below students describe the intervention as providing an opportunity to step back from academic pressure, and encouraging self-care practices.

*“I even made sure that indulged in self-care as well which I don’t often have time for.”* (VR group).

*“Watching the videos was a small time that I could switch off and relax between studying.”* (Video group).

4. Broader and sustained impact on well-being.

This theme captures the extended and cumulative effects of VR use, in addition to the immediate effects on students’ well-being, including improved sleep quality, reduced stress, improvements in productivity and the development of sustainable self-regulation practices.

Transferable skills: this subtheme reflects the development of psychological techniques such as mindfulness, emotional regulation, and self-soothing techniques, which extended beyond the VR sessions themselves. Notably, some indicated that these practices became easier and more automatic over time, suggesting the potential for longer-term behavioural change.

Here, students describe broader and more sustained improvements in their well-being and daily functioning

*“I’m getting more work done*,* getting more sleep*,* and waking up feeling less suffocated.”* (VR group).

*“I feel more relaxed*,* I feel capable and in control […] I also feel I have been sleeping better and had lower than usual stress level.”* (VR group).

*“This experience has been very useful in developing skills to relax*,* comfort myself*,* quieten my thoughts and be mindful.”* (VR group).

*“It’s forced me to be mindful which I will practise moving forwards.”* (VR group).

5. Conditional and limited impact of non-immersive video.

In contrast to the VR experience, responses from the Video group highlighted a more variable and often limited impact, with many students describing the benefits as modest, short-lived, or negligible.

Although some students reported feeling calmer or more relaxed after watching the Videos, these effects were typically described as less intense and less enduring. A central factor underpinning this difference was the perceived lack of immersion, with participants frequently attributing reduced effectiveness to the constraints of viewing content on a phone.

For many, the Videos functioned primarily as a brief opportunity to pause or focus on breathing, rather than producing a substantive shift in emotional or cognitive state. Several participants also noted that the short duration and limited frequency of the sessions may have further constrained their impact.

*“I’ve definitely felt calmer after watching the Video.”* (Video group).

*“Not really had much effect on me personally.”* (Video group).

*“I didn’t feel that much difference because each Video was only 7 minutes and we only watched one a day.”* (Video group).

*“I think due to the lack of immersion*,* I didn’t feel much change after watching the Videos.”* (Video group).

## Discussion

A novel hypnotherapy-based immersive VR intervention was trialled in a student population in a central London healthcare university, compared with the same experience on video.

### Summary of findings

There was a significant overall reduction in perceived stress from baseline to follow-up across both groups. Within-group effect sizes were moderate for VR and small for Video. Both interventions significantly improved momentary affect (reduced stress, anxiety, sadness; increased calm and happiness). However, VR produced significantly greater immediate improvements than Video for stress, anxiety, calmness, and happiness. These benefits were strongest in early sessions.

VR participants reported substantially higher immersion than Video participants, and immersion was moderately correlated with emotional improvements, particularly in the VR group.

Among participants completing the intervention, stress reduction remained significant, with stronger effects in VR. Only VR participants showed statistically significant reductions in perceived stress at follow-up.

Participants strongly preferred VR, reporting greater perceived benefit, calmness, and likelihood of future use. Qualitative analysis showed 89% of VR users reported experiencing a positive effect on their well-being.

The significant decrease in pre-trial-to-follow-up levels of perceived stress in VR participants reflects findings that have been reported in the literature for students^16,26,27^. There was little evidence for a benefit of the VR application on follow-up measures of wellbeing and mood.

### Mechanistic pathways: role of immersion and presence

Both VR and Video group participants showed some pre-to-post session improvement in measures. This is likely due to the Video being an active control. Overall, VAS change scores were significantly greater for participants experiencing VR than those receiving the Video intervention for measures for stress, anxious, calm and happy. The concept of presence in virtual reality describes “the extent to which one feels present in the mediated environment, rather than in the immediate physical environment”^41^. Tham et al., (2018) further reported that a participant’s sense of presence is enhanced by various features of the virtual environment^42^. Participants in the VR group may have felt more present in the experience. The association between pre-to-post session change on VAS measures and level of immersion was statistically significant for all VAS measures in the VR group, while being significant for only stress and calm in the Video group.

Figure S3a–d display adjusted pre-to-post session mean (CI) change scores over the study for VR Headset (VR) and Video intervention study participants according to session for those participants who adhered to their intervention (per-protocol). The figures indicate that differences in change scores were more likely in early sessions (Day 1, Day 2) rather than later, perhaps suggesting the novelty of the VR intervention may have had a differentially positive impact on momentary affective states.

### Clinical implications for student mental health

VR participants reported reduced perceived stress from pre-intervention to follow-up at two weeks, while there were no significant changes in the Video group, echoed by the therapeutic effect that students described. This appears to indicate the superiority of the immersive VR experience in producing sustained improvements in stress levels, consistent with the findings of similar studies^16–18^. Xu et al.^20^ found that VR reduced stress experienced by young adults and students and was an accessible and affordable tool. In a randomised crossover trial, Kim et al., (2021) found VR relaxation effective in reducing stress^43^. In order to investigate longitudinal effects of VR, our study included additional follow-up at two weeks, demonstrating that the effects for perceived stress can be sustained.

Perhaps surprisingly, both VR and Video group participants showed pre-to-post session improvements in cybersickness scores on the SSQ. Several studies offer potential explanations for this effect. Bouchard et al. argued that there is an overlap between symptoms of anxiety and unwanted side effects of VR, which may increase SSQ scores^44^. Given that the mean baseline perceived stress (PSS-10) scores for both VR and Video group participants were within the moderate stress range, pre-existing or anticipatory anxiety may contribute to the explanation of why cybersickness scores were high before the intervention and that seated delivery and stress reduction effects of both interventions mediated cybersickness. This is an important clinical finding for VR research confirming that cybersickness is not an inevitable adverse effect of VR.

In the qualitative data, 42 (of 47) participants receiving VR reported experiencing a therapeutic effect on their emotional well-being. In the Video group, only 17 of 50 reported this, reflected in participants’ written comments, which were more positive about the experience if they received VR. Several participants in the Video group referred to the lack of immersion in the experience as being one of the reasons they did not think there was a therapeutic effect of watching the Video. This appears to add weight to the importance of the concept of presence that is provided by VR. Various comments in the VR group referred specifically to exams and university studies and the beneficial impact that VR has had in feeling positive and productive.

### Strengths

Our study underwent several stages of refinement as part of the co-production of the VR intervention with students that highlighted the importance of clear written instructions of what to expect, contributions on colour design, audio and visual effects. This was a strength of the study. Importantly our experimental design allowed us to isolate the immersive variable and provide methodological stringency by controlling for content exposure in our Video control condition unlike traditional comparison conditions such as waitlist or treatment-as-usual.

This study included simultaneous assessment of both dispositional constructs (perceived stress, wellbeing, depression) and momentary affective states providing a multilevel validation of intervention efficacy with the addition of rich qualitative data analysis.

Our findings have important practical implications for real-world implementation and intervention scalability. The provision of a seven-minute daily intervention across five consecutive days is feasible within university timetables and there is potential for peer-led support, partnership with student unions, personal tutoring systems, library services and student wellbeing services within higher education institutions. In addition, VR headsets are relatively inexpensive and have established reliability, accessibility and engagement for people across the lifespan.

### Limitations and generalisability

The study was based at one university and neither the participants nor researchers were completely blind to the intervention. As all students were invited to participate, it is possible that students who were more enthusiastic about VR and students with more mental health concerns may have volunteered. It is important to consider whether the results of this study are generalisable as the majority of the students who participated were female with a mean age of nearly 22 in a university setting. Notably the university has an internationally diverse student population. The study results reflect only the self-report survey results from the students who were participating. Physiological measures of stress, such as heart rate and blood pressure, were not taken. It is possible that students in the VR group may have been more enthusiastic about their allocated intervention if it was their first experience with VR. Additional objective measurements alongside the subjective experience of the students may add weight to the results. While this study measured stress in the students before and after each intervention and two weeks after, it did not consider whether there was a measurable effect on examination results. Baseline imbalance in stress scores may have influenced observed changes.

The VR experiences were created for participants to use one each day for five consecutive days. However, this does not provide information on whether less frequent use or using VR whenever students feel like it would produce similar effects. The two-week follow-up period was relatively short; a longer-term use of the intervention and follow-up would be of value to establish clinical efficacy over time and to determine whether stress reduction is sustained or diminished over a longer time period. We also acknowledge that the qualitative data was elicited through responses to free-text open questions, which is limited in its richness and that more detailed qualitative interviews and data analysis would be useful. In addition, qualitative interviews could provide opportunities to explore attrition and the reasons for non-attendance to elicit insights into the potential drop-out mechanisms for the 19% of participants who did not complete the 5-day intervention. Future studies should embed this into the study design to enhance real-world implementation and scalability.

All researchers were trained in the procedural elements of the study which were designed to be clear, straightforward and consistent to maintain procedural fidelity. However, there were no formal measures recorded and there is scope to Video record researchers to ascertain adherence consistency and fidelity coding in future studies to enhance methodological rigour.

No formal economic analysis was conducted as part of this study, as the main effects were limited to stress reduction. The cost implications of VR versus traditional interventions could be fully evaluated and considered for future studies. Cost per quality-adjusted life year (QALY) or a cost-effectiveness threshold assessment could inform institutional adoption decisions in future.

### Future research directions

A larger Randomised Controlled Trial with additional control conditions across different sites could explore further improvements to the VR environment, including user preferences for location, timing, access and engagement would be beneficial. There is scope to further explore the potential psychological and physiological mechanisms across selected cognitive, psychological and behavioural outcomes.

### Summary

This randomised controlled trial of a novel VR intervention co-designed with students demonstrated that a clinically-informed immersive VR experience significantly reduced stress at follow up and was well tolerated in a student population. No side effects were reported. The control group also experienced benefits of the experience without immersion, demonstrating an active control, but these improvements were not as marked or sustained. This study adds to the emerging literature focussing on VR in the student population^19–21,45,46^ and builds on an earlier pilot study^26^ with the addition of a control arm. Participants in both arms reported an improvement in stress and anxiety but in the VR group these were more pronounced, suggesting that there is something about the immersive experience that enhances the effect of the experience.

## Conclusions

This study demonstrated that a series of Video/ audio multisensory experiences aimed at reducing stress in students had a significant positive result in both the phone/audio and immersive VR group. The VR results were significantly better at follow up with improvement sustained at 2 weeks for levels of perceived stress. The qualitative results added depth and richness to our understanding of individual experiences which highlighted the unique benefits of immersion. At a time when there is an urgent need for more mental health interventions in the student population, it seems that this clinically-informed VR intervention offers an innovative, engaging, sustainable and accessible solution for healthcare students experiencing stress in a higher education setting.

## Supplementary Information

Below is the link to the electronic supplementary material.

Supplementary Material 1

## Data Availability

The data that support the findings of this study are available on request from the corresponding author, AO’B.

## Declarations

### Transparency declaration

The manuscript is an honest, accurate, and transparent account of the study being reported; that no important aspects of the study have been omitted; and that any discrepancies from the study as planned (and, if relevant, registered) have been explained.
