# Supplementary material for: A controlled trial of a co-designed virtual reality experience to support wellbeing in students
Source: Sci Rep. 2026 Aug 7;16:36941. doi: 10.1038/s41598-026-61827-6 (PMC13736701; doi:10.1038/s41598-026-61827-6)
Supplement: Supplementary file 1 [file 41598_2026_61827_MOESM1_ESM.docx]

## Supplementary Table and Figures

| **Table S1**. Associations (partial correlations) between (adjusted) mean pre-to-post session change on VAS measures and levels of intervention immersion, post-session SSQ score and (adjusted) pre-to-post session change on the SSQ for VR Headset (VR) and Video intervention participants. | | | | | |
| --- | --- | --- | --- | --- | --- |
|  | Stress | Anxious | Sad | Calm | Happy |
|  | r (CI) | r (CI) | r (CI) | r (CI) | r (CI) |
| VR participants |  |  |  |  |  |
| Immersion | 0.42**  (0.07,0.70) | 0.52***  (0.21,0.74) | 0.29*  (0.04,0.52) | 0.64***  (0.49,0.78) | 0.50*** (0.26,0.71) |
| SSQ total (post-session) | -0.13  (-0.41,0.20) | -0.05  (-0.37,0.31) | -0.05  (-0.35,0.19) | -0.07  (-0.36,0.38) | -0.01  (-0.37,0.38) |
| SSQ change (adjusted) | -0.13  (-0.37,0.11) | -0.09  (0.15,-0.37) | -0.14  (-0.38,0.05) | -0.19  (-0.46,0.13) | -0.19  (-0.47,0.07) |
| Video participants |  |  |  |  |  |
| Immersion | 0.48**  (0.18,0.68) | 0.27  (0.01,0.48) | 0.09  (-0.27,0.39) | 0.43** (0.12,0.68) | 0.17  (-0.20,0.54) |
| SSQ total (post-session) | -0.27  (-0.53,0.06) | -0.01  (-0.29,0.28) | -0.06  (-0.39,0.24) | -0.08  (-0.38,0.28) | 0.13  (-0.20,0.43) |
| SSQ change (adjusted) | -0.33*  (-0.57,-0.06) | -0.12  (-0.37,0.17) | -0.16  (0.15,-0.45) | -0.18  (-0.48,0.17) | -0.08  (-0.43,0.24) |
| Note: Mean pre-to-post-session change on VAS measures were adjusted for pre-session scores. SSQ = Simulator sickness questionnaire; pre-to-post session change on the SSQ was adjusted for pre-session score. *p < 0.05, **p < 0.01, ***p < 0.001. | | | | | |

**Figures S1a-S1c**. Perceived stress, wellbeing and mood scores at baseline and follow-up for (VR) and Video intervention participants (per-protocol approach; **S1a** = Perceived Stress Scale (PSS); **S1b** = Warwick-Edinburgh Mental Wellbeing Scale (WEMWBS); **S1c** = Patient Health Questionnaire-2 (PHQ-2)). Value labels represent mean values. Error bars are 95% confidence intervals.

**Figure S2**. Adjusted pre-to-post session mean change scores over the study for VR Headset (VR) and Video intervention study participants (per-protocol approach). Value labels represent mean values. Error bars are 95% confidence intervals. Asterisks indicate significant differences between study conditions: *p < 0.05, **p < 0.01, ***p < 0.001.

**Figure S3a-3d**. Adjusted pre-to-post session mean (CI) change scores over the study for VR Headset (VR) and Video intervention study participants according to session (per-protocol approach; **S3a** = Stress; **S3b** = Anxious; **S3c** = Calm; **S3d** = Happy). Value labels represent mean values. Error bars are 95% confidence intervals. Asterisks indicate significant differences between study conditions: *p < 0.05, **p < 0.01, ***p < 0.001.
